# Altered hypoxia‐inducible factor–1*α* (HIF‐1*α*) signaling contributes to impaired angiogenesis in fetal lambs with persistent pulmonary hypertension of the newborn (PPHN)

**DOI:** 10.14814/phy2.13986

**Published:** 2019-01-31

**Authors:** Kartikeya Makker, Adeleye J. Afolayan, Ru‐Jeng Teng, Girija G. Konduri

**Affiliations:** ^1^ Department of Pediatrics University of Florida College of Medicine Jacksonville Florida; ^2^ Department of Pediatrics Cardiovascular Research Center and Children's Research Institute Medical College of Wisconsin Milwaukee Wisconsin

**Keywords:** Endothelial dysfunction, Glycolysis, reactive oxygen species

## Abstract

Previous studies in adult pulmonary hypertension reported that increased hypoxia‐inducible factor–1*α* (HIF‐1*α*) signaling contributes to pulmonary vascular remodeling. However, alterations in endothelial HIF‐1*α* signaling and its contribution to impaired angiogenesis in persistent pulmonary hypertension of the newborn (PPHN) remain unclear. We investigated the hypothesis that HIF‐1*α* levels are increased in lung endothelial cells in PPHN and contribute to impaired angiogenesis function. We examined HIF‐1*α* expression and promoter activity in the isolated pulmonary artery endothelial cells (PAEC) from fetal lambs with or without PPHN induced by prenatal ductus arteriosus constriction. We measured the levels of HIF‐1*α* downstream targets, vascular endothelial growth factor (VEGF) and glycolytic protein, hexokinase 2 (Hek‐2) in PAEC from PPHN, and control lambs. We examined the effect of small interfering‐RNA (siRNA) mediated knockdown of native HIF‐1*α* on VEGF expression and in vitro angiogenesis function of PPHN‐PAEC. HIF‐1*α* protein levels were higher in the isolated PAEC from PPHN‐lambs compared to controls. HIF‐1*α* promoter activity and Hek‐2 protein levels were higher in PPHN. VEGF protein levels and in vitro angiogenesis function were decreased in PAEC from PPHN lambs. HIF‐1*α* silencing significantly increased the expression of VEGF and improved the angiogenesis function of PPHN PAEC. Aberrant HIF‐1*α* signaling contributes to endothelial dysfunction and decreased angiogenesis in PPHN.

## Introduction

Persistent pulmonary hypertension of the newborn (PPHN) occurs when the pulmonary vascular resistance (PVR) fails to decrease at birth, leading to decreased blood flow to the lungs and impaired gas exchange (Fox et al. 1977). The vascular dysfunction evolves antenatally and interferes with normal adaptation of pulmonary circulation at birth. Infants affected by PPHN develop significant hypoxemia, which increases the risk of mortality and long‐term disabilities (Walsh‐Sukys et al. 2000; Konduri et al. 2007b). Recent advances in neonatal care, including the use of inhaled nitric oxide (iNO) and other vasodilators have improved the outcome in PPHN infants. However, up to 30% of neonates affected by PPHN fail to respond to this therapy, requiring invasive life support measures (Konduri et al. 2004). The mechanisms responsible for the impaired vasodilator responses in PPHN remain unclear.

HIF‐1*α* is a basic helix‐loop‐helix PAS domain containing protein and is a master transcriptional regulator of cellular and developmental response to hypoxia. HIF‐1*α* enables cells to respond to decreased oxygen (O_2_) availability (Koulmann et al. 2006; Resnik et al. 2007) by upregulating the transcription of genes involved in the hypoxia response, including vascular endothelial growth factor (VEGF) and hexokinase II (Hu et al. 2003; Semenza 2005; Marín‐Hernández et al. 2009; Rey and Semenza 2010).

The transcription factor HIF consists of a labile *α* subunit and a stable *β* subunit. In the presence of O_2_, prolyl hydroxylase enzyme isoforms hydroxylate HIF‐1*α* subunits on the conserved prolyl residues. Hydroxylated HIF‐1*α* is then polyubiquitinated and degraded by a ubiquitin ligase that contains the pVHL tumor suppressor protein (Park et al. 2010). Under hypoxic conditions, or in the absence of pVHL, HIF accumulates in its active form and transcriptionally activates multiple genes involved in acute or chronic adaptation to hypoxia, like erythropoietin (EPO) and VEGF (Park et al. 2010). HIF‐1*α* is overexpressed and promotes cell proliferation and formation of abnormal blood vessels in cancer cells (Semenza 2003, 2005; Rey and Semenza 2010).

Elevated HIF‐1*α* protein level was previously reported in the experimental models of PAH in mice and fawn hooded rats due to normoxic stabilization of this protein (Park et al. 2010; Rehman and Archer 2010; Bishop and Ratcliffe 2015). A previous study in the fetal lamb model of PPHN demonstrated elevated HIF‐1*α* levels in the pulmonary artery smooth muscle cells when cultured under normoxic conditions and showed that HIF‐1*α* overexpression leads to pulmonary artery smooth muscle cell proliferation (Wedgwood et al. 2015). However, alterations in HIF‐1*α* signaling in the pulmonary artery endothelial cells and its role in altered adaptation remain unclear. We previously reported that a decrease in angiogenesis in the lung occurs due to oxidative stress in PPHN (Teng et al. 2009). Oxidative stress has been implicated as a mechanistic factor in the pulmonary vascular dysfunction in PPHN (Konduri et al. 2003, 2007a; Wedgwood et al. 2005). Whether HIF‐1*α* levels increase in PAEC and contribute to impaired angiogenesis in PPHN remain unknown. We therefore *hypothesized* that HIF‐1*α* is overexpressed in PAEC and contributes to impaired angiogenesis function of PAEC in PPHN. We investigated our hypothesis in PAEC and lung tissue from lambs with PPHN induced by prenatal ductus arteriosus constriction. Our present studies demonstrate that HIF‐1*α* levels are increased in PAEC and this aberrant HIF‐1*α* signaling contributes to impaired angiogenesis function of PAEC in PPHN.

## Materials and Methods

### Creation of PPHN lamb model

Our study was approved by the Institutional Animal Care and Use Committee of the Medical College of Wisconsin and conformed to NIH guidelines for animal use. Constriction of the fetal ductus arteriosus was performed at 128 ± 2 days of gestation (term ≈ 144 days) and maintained for 8 days in utero, as described previously (Konduri et al. 2003, 2007a). Control fetal lambs had thoracotomy performed without constriction of the ductus arteriosus. After 8 days of ductal constriction, the fetal lambs were delivered by Cesarean section, and right ventricular peak systolic pressures (RVSP) were measured by Millar MicroTip catheter with a pressure transducer (ADI Instruments, Colorado Springs, CO). Fetal lungs were harvested for the isolation of pulmonary arteries and PAEC and for lung histology, as outlined below.

### Histology and right ventricular systolic pressure measurement

**Figure 1:**
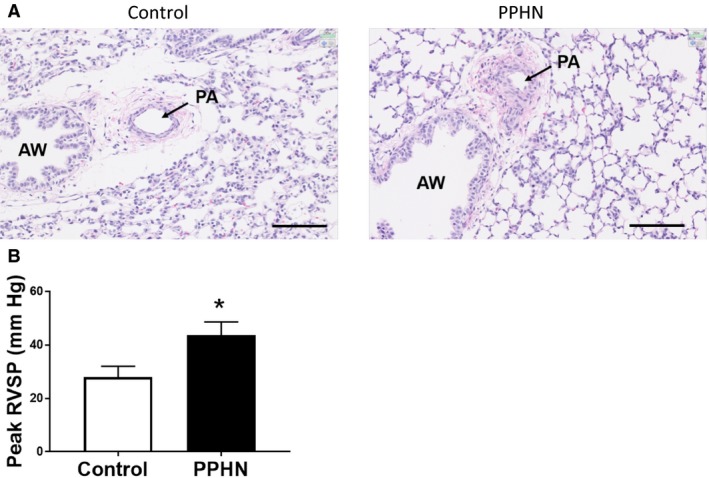
Lung histology and right ventricular systolic pressure (RVSP) in control and PPHN lambs. Lung histology shows medial thickening and narrowing of the lumen of pulmonary artery (PA) adjacent to the airway (AW) in a representative section of PPHN lamb lung compared to control lamb (A). Lung was inflation fixed with 10% formaldehyde and paraffin section was stained with hematoxylin and eosin. The peak right ventricular systolic pressure, measured with a Millar catheter containing a pressure transducer, was significantly higher in PPHN lambs compared to control lambs (B). * indicates *P* < 0.05 by unpaired T test for *n* = 3 lambs in each group.

One lobe of the fetal lung was inflation fixed at 10 cm H_2_O pressure with 10% formaldehyde infused via the lobar bronchus. Lung tissue was then fixed in 10% formalin for 48–72 h and subjected to paraffin embedding and sectioning to thickness of five microns. The sectioned slides were stained with hematoxylin and eosin (H&E) and imaged using NanoZoomer 2.0‐HT and NDP scan. Representative images are shown in Figure 1.

Right ventricle was cannulated with 3Fr Millar MicroTip catheter with pressure transducer at the tip, connected to appropriate amplifier with data acquisition system (ADI instruments). RVSP measurements were obtained from three control and three PPHN lambs.

### Isolation of PAEC

**Figure 2:**
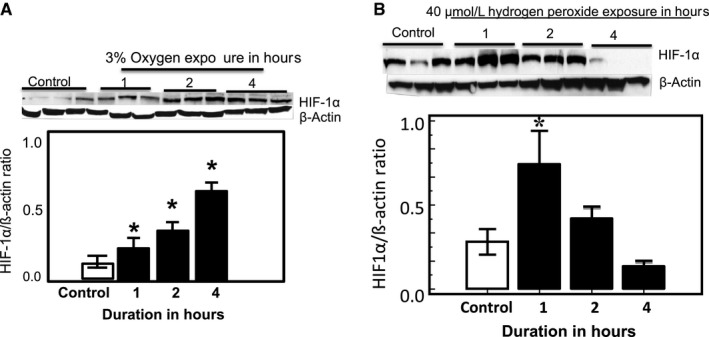
Hypoxia and H_2_O_2_ induce HIF‐1*α* expression in normal pulmonary artery endothelial cells (PAEC). Representative immunoblots of HIF‐1*α* protein levels in response to: (A) 3% O_2_ and (B) 40 *μ*mol/L hydrogen peroxide. * indicates *P* < 0.05 from normoxic (A) or untreated (B) controls. (*n* = 6)

PAECs were isolated and characterized using techniques described previously (Konduri et al. 2003, 2007a). Briefly, endothelial cells were isolated from third to fifth generation pulmonary arteries with 0.25% collagenase type A (Roche Molecular Bio‐chemicals, Indianapolis, IN) and cells were grown in Dulbecco's modified Eagle's medium (DMEM) with 20% fetal calf serum (FCS). Endothelial cell identity was verified by the presence of factor VIII antigen and acetylated low‐density lipoprotein uptake as stated earlier (Konduri et al. 2003). PAEC isolated from at least four different fetal lambs were used for each group. PAEC from control and PPHN lambs between passages three and six were used at the same time in parallel for each experiment for appropriate comparison. We previously reported that PAEC from PPHN lambs maintain their altered phenotype until at least passage six (Konduri et al. 2003, 2007a). All the experiments were carried out under normoxic conditions except for the experiment where the investigators specifically investigated the effect of 3% hypoxia on HIF‐1*α* protein levels in control PAEC (result 1, Fig. 2).

### Western blot analyses

PAEC were grown to near confluence and washed twice with ice‐cold HBSS. The lungs and PAEC were lysed in modified MOPS (3‐[*N*‐morpholino] propane sulfonic acid) buffer. The cell lysate was sonicated, and cell debris was removed by centrifugation at 9600 g. Protein content of the lysate was determined by bicinchoninic acid method (BCA). Proteins were separated by SDS‐PAGE, transferred to nitrocellulose membranes, and were blotted with specific polyclonal antibodies for HIF‐1*α*, HIF‐1*β*, HIF‐2*α*, Hek‐II, VEGF, and *β*‐actin overnight at 4°C. The membranes were blotted with horseradish peroxidase (HRP)‐conjugated anti‐mouse or anti‐rabbit IgG antibody (1:9,000; Bio‐Rad) and exposed to CL‐XPosure films (Pierce) after treatment with SuperSignal West Pico (Pierce). The signals were analyzed with ImageJ and normalized to the expression of the *β*‐actin as loading control.

### Promoter luciferase assays in PAEC

PAEC from PPHN and control lambs were transfected with plasmids containing dual luciferase system with HIF reporter (Cignal HIF reporter assay, SA Biosciences, Frederick, MD) encoding a firefly luciferase gene under the control of minimal CMV promoter fused with tandem repeats of hypoxia response element and a constitutively expressing Renilla construct, which served as internal control. Appropriate negative controls were used with noninducible luciferase construct and Renilla luciferase construct. In other reporter assays, PAEC from PPHN and control lambs were transfected with 1–2 *μ*g of pcGL4.10‐VEGFR2 promoter (−500 to −1), 4 *μ*g of pcDNA3, and 50 ng of the Renilla luciferase expression plasmid pRL‐CMV (Promega) by using FuGENE® 6 transfection reagent (Promega) according to the manufacturer's instructions. After 24 h, luciferase activity of the cell extract was measured by the Dual Luciferase Assay System according to the manufacturer's instructions and a Berthold Technologies luminometer.

### HIF‐1*α* knockdown

Silencing RNA (siRNA) for bovine HIF‐1*α* (sc‐270450 Santa Cruz Biotechnology) was used for transfection with Lipofectamine RNAiMAX Transfection Reagent (Invitrogen) following standard protocol. SiRNA (30pM), or nonsilencing RNA, was mixed with 500 *μ*L Opti‐MEM‐I reduced serum medium in six‐well plates. Lipofectamine RNAiMAX (5 *μ*l) was then added and incubated for 20 min at room temperature. PPHN‐PAEC (3 × 10^5^) suspended in antibiotic free culture medium was added into the siRNA mixture for 72 h at 37°C in a humidified CO_2_ incubator. Subsequently HIF‐1*α*, HekII, and VEGF protein expression was assessed by immunoblotting.

### In vitro angiogenic activities

In vitro angiogenesis was studied as previously reported (Teng et al. 2009, 2013) by capillary tube formation in Matrigel and monolayer scratch recovery assays.

### Tube formation assay

PAEC (4 × 10^4^/well) were transfected with HIF‐1*α* siRNA or scramble RNA and suspended in 200 *μ*l of culture medium (2% FCS) were plated into individual wells containing Matrigel. Capillary‐like structures were identified and the length of individual tubes was measured 6 h after plating by one of the coauthors blinded to the treatment under 10x (objective) magnification (Teng et al. 2009, 2013).

### Scratch recovery assay

PAEC were grown to confluence in 12‐well plates. Scratch lines were created by 1‐mL pipette tip and the wells were gently rinsed with Hanks's Balanced Salt Solution to remove the detached cells. The cells were serum starved in DMEM with 2% FCS for 60 min and the medium was changed back to DMEM with 20% FCS. The distance of the gap between the frontlines of recovery was measured for comparisons (Teng et al. 2009, 2013).

### Statistical analyses

Data are shown as means ± SEM. Student's *t*‐test was used for normally distributed data, and Mann–Whitney *U*‐test was used for data that did not pass the normality test for comparison between the two groups. Data were analyzed with MedCalc. A *P* < 0.05 was considered significant.

## Results

1. PPHN lambs show evidence of medial hypertrophy of pulmonary arteries and increased RVSP: Compared to control lambs, PPHN lambs show medial hypertrophy of pulmonary arteries, and narrowing of the arterial lumen on H&E staining (Fig. 1A). PPHN lambs also show significantly higher RVSP compared to control lambs, with an average increase of 15 mmHg (Fig. 1B). These data show evidence of elevated pulmonary artery pressure and vascular remodeling in our fetal lamb model of PPHN.
2. Hypoxia and exogenous hydrogen peroxide (H_2_O_2_) increase HIF‐1*α* protein levels in normal PAEC. These studies were done to verify that HIF‐1*α* in the isolated fetal lamb PAEC shows the expected responses to physiologic stimuli. We investigated the response of control PAEC to hypoxia by exposing cells to either 21% or 3% O_2_. In PAEC exposed to 21% O_2_, the expression of HIF‐1*α* protein was barely detectable. Exposure to 3% O_2_ increased HIF‐1*α* protein expression within 1 h following hypoxia exposure with a peak response at 4 h, followed by decrease in levels (Fig. 2A). Similarly, exogenous H_2_O_2_ (40 *μ*mol/L) also increased HIF‐1*α* protein expression, which peaked at 1 h and returned to baseline at 4 h following H_2_O_2_ treatment (Fig. 2B). These findings are consistent with previous reports and suggest that hypoxia and ROS are capable of inducing HIF‐1 *α* protein expression in normal fetal lamb PAEC.
3. Expression of HIF‐1*α*, HIF‐1*β,* and HIF‐2*α* are increased in PPHN‐PAEC. Previous studies from our lab have demonstrated increased ROS formation in PPHN‐PAEC (Konduri et al. 2007a; Teng et al. 2009; Afolayan et al. 2012). We investigated HIF protein levels in control and PPHN‐PAEC grown under normoxic condition by Western blots. HIF‐1*α* protein was significantly increased in the isolated PAEC from PPHN compared to control lambs (Fig. 3A). Similarly, levels of HIF‐1*β* and HIF‐2*α* proteins were also increased in PPHN‐PAEC (Fig. 3B and C).

**Figure 3:**
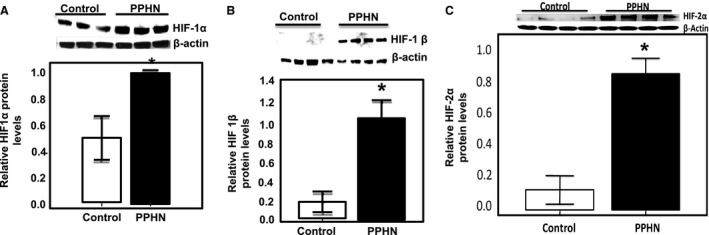
Protein levels of HIF‐1*α*, and HIF‐1*β*, HIF‐2*α,* were all increased in PPHN. Representative immunoblots of corresponding proteins in normal and PPHN PAEC depicting (A) HIF‐1*α*, (B) HIF‐1*β*, and (C) HIF‐2*α*. * *P* < 0.05 from controls (*n* = 4)
4. HIF‐1*α* activity is significantly increased in PPHN. We then investigated the functional effects of increased HIF‐1*α* protein levels in PPHN‐PAEC by quantifying HIF‐1*α* promoter‐reporter activity and the expression of its target gene, hexokinase‐II (Hek‐II). We observed a significant increase in HIF‐1*α* promoter activity, as assessed by dual luciferase assay (Fig. 4A) which measures the binding of HIF‐1*α* to the consensus hypoxia response element sequence. To verify the downstream effect of increased HIF‐1*α* activity, we blotted for protein levels of Hek‐II, a key glycolytic protein and target of HIF‐1*α*. We found an increase in HeK‐II levels in PPHN‐PAEC (Fig. 4B). We additionally observed that PPHN‐PAEC transfected with siRNA targeted against native HIF‐1*α* protein show decrease in Hek‐II protein levels (Fig. 4C). These findings suggest that increased HIF‐1*α* activity in normoxia contributes to HIF‐1*α*–dependent glycolytic gene expression in PPHN‐PAEC.

**Figure 4:**
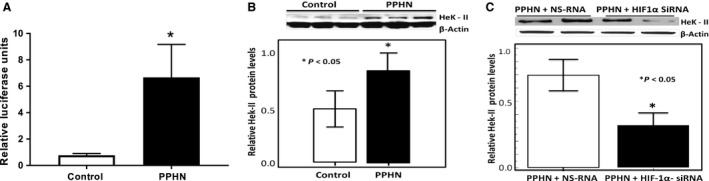
Promoter activity for HIF‐1*α* (A) and hexokinase‐II (HeK‐II, B) in control and PPHN endothelial cells. HIF‐1*α* promoter activity and HeK‐II protein were increased, indicating stabilization of HIF‐1*α* protein in PPHN. Knockdown of HIF‐1a in PPHN cells using siRNA decreased the HeK‐II levels (C) (*n* = 5, and * indicates *P* < 0.01 for HIF‐1*α* promoter from control (A) and *P* < 0.05 from control (B) or PPHN+NS‐RNA (C)).
5. HIF‐1*α* inhibition increased VEGF protein levels in PPHN‐PAEC: Although HIF‐1*α* is a major transcription factor involved in hypoxia mediated angiogenesis response, the effects of aberrant HIF‐1*α* signaling during normoxia remain unclear. Although HIF‐1*α* levels were increased in PPHN, we found decreased VEGF expression in PPHN‐PAEC when compared to controls (Fig. 5A). We next determined whether the decrease in VEGF protein leads to an increase in the levels of its functional receptor, VEGFR2. We quantified VEGFR2 promoter activity in control and PPHN‐PAEC using a dual luciferase assay. We observed robustly increased VEGFR2 receptor promoter activity in PPHN‐PAEC (Fig. 5B). This is associated with the increase in VEGFR2 protein level (Fig. 5C), suggesting a feedback compensation for the decreased VEGFA protein levels. To determine the contribution of aberrant HIF‐1*α* signaling to VEGF downregulation, we transfected PPHN‐PAEC with siRNA targeting native HIF‐1*α* in PPHN‐PAECs and measured VEGFA and HIF‐1*α* protein levels by Western blots. HIF‐1*α* silencing decreased the HIF‐1*α* levels as expected (Fig. 5D) and significantly increased VEGFA protein levels in PPHN‐PAEC compared to nonsilencing siRNA used as controls (Fig. 5E). These findings suggest that aberrant HIF‐1*α* signaling is associated with decreased VEGF levels under normoxic condition.

**Figure 5:**
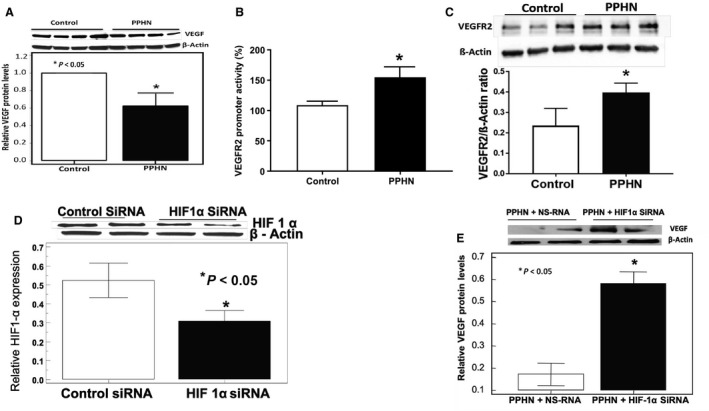
VEGF protein levels in control and PPHN PAEC and the effects of HIF‐1*α* knockdown. (A) Representative immunoblot and summarized data of VEGF expression in control and PPHN PAEC, (B) Increased VEGFR2 promoter activity in PPHN, (C) Increased VEGFR2 protein level in PPHN, (D) Immunoblot and summary data for HIF‐1*α* after its knockdown with siRNA, and (E) HIF 1‐*α* siRNA transfection effects on VEGF protein level compared to nonsilencing RNA (NS‐RNA). *P < 0.05 from controls or PPHN+NS‐RNA (*n* = 5)
6. HIF‐1*α* knockdown improves angiogenesis function of PAEC in PPHN. In the Matrigel assay, tube formation was impaired in PAEC from PPHN lambs, compared to control cells (Fig. 6A – a and b), similar to our previous report (Teng et al. 2009). Tube formation significantly increased after HIF1‐*α* knockdown in PPHN‐PAEC (Fig. 6A – d and e) (NS‐siRNA 100.0 ± 7.3% vs. HIF1‐*α* siRNA 139.6 ± 6.8%; *n* = 20, *P* < 0.05). Cell migration into scratch margins was decreased for PPHN‐PAEC compared to control PAEC (Fig. 6B – a and b), as we reported previously (Teng et al. 2009, 2013). Cell migration was significantly increased by HIF‐1*α* knockdown in PPHN‐PAEC as depicted by a decrease in gap margin (Fig. 6B – d and e) (NS‐SiRNA 639.36 ± 19.8 *μ*m vs. HIF‐1*α* siRNA 482.16 ± 33.52 *μ*m; *n* = 10, *P* < 0.05).

**Figure 6:**
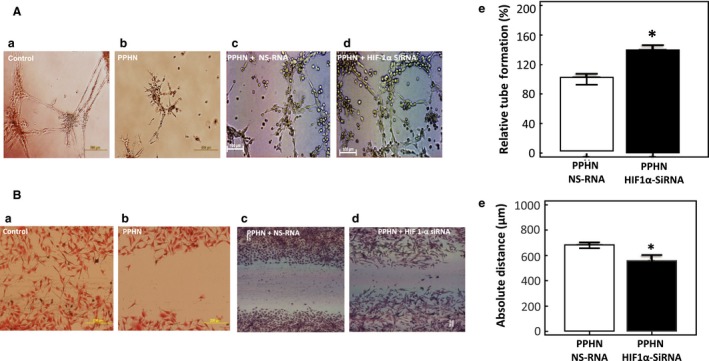
Effect of HIF 1‐*α* knockdown on in vitro angiogenesis function of PAEC. (A) Tube formation in Matrigel for control (a) and PPHN (b) PAEC. Tube formation was impaired in PPHN cells. The effect of nonsilencing RNA (c) or HIF‐1*α* siRNA transfection on tube formation by PPHN cells. Tube formation by PPHN PAEC was improved by HIF‐1*α* siRNA treated cells (d & e). * *P* < 0.05 from controls (*n* = 20). (B) Cell Migration by control and PPHN PAEC and effect of HIF‐1*α* siRNA transfection. Cell migration into scratch margin was impaired for PPHN cells (b) compared to controls (a). HIF‐1*α* knockdown with siRNA improves cell migration into scratch margin by PPHN cells (d & e) compared to nonsilencing RNA treated PPHN cells (c). *P < 0.05 from controls (*n* = 20).

## Discussion

In this study, we found that HIF‐1 *α* is strongly upregulated in the PAEC in a fetal lamb model of PPHN. These data add to the previous report of increased HIF‐1*α* levels in vascular smooth muscle cells in PPHN and models of adult PAH (Bonnet et al. 2006; Wedgwood et al. 2015). We also made a novel observation that increased HIF‐1*α* levels impair VEGFA expression and angiogenesis function of PAEC in PPHN.

PPHN is characterized by the presence of increased ROS production and oxidative stress (Brennan et al. 2003; Konduri et al. 2003, 2007a), which contribute to the vascular remodeling and elevated pulmonary artery pressure we observed in our model. ROS were reported to inhibit the prolyl hydroxylases that target HIF‐1*α* for degradation (Scortegagna et al. 2003; Semenza 2003, 2005). We observed that HIF‐1*β* and HIF‐2*α* levels were also increased in PPHN‐PAEC. This is consistent with increased HIF‐1*α* promoter activity since both *α* and *β* subunits dimerize to form the active form of HIF‐1*α*. HIF‐2*α* also dimerizes with HIF‐1*β* to promote transcription of several genes that may be involved in PAH pathogenesis (Semenza 2003, 2005).

Our data demonstrated the responsiveness of control PAEC to hypoxia and ROS with appropriate changes in HIF‐1*α* expression. On exposure to 3% hypoxia and 40 *μ*mol/L hydrogen peroxide, we observed a significant increase in HIF‐1*α* levels. In the hydrogen peroxide exposed control PAEC, the increase in HIF‐1*α* at 1 h was followed by a decrease in baseline at 2 h and 4 h. We hypothesize that catalase, or other antioxidant systems, in control PAEC are intact and lead to a decrease in oxidative stress and subsequent normalization of HIF‐1*α* levels, in contrast to the effect of hypoxia.

Decreased blood vessel density in PPHN‐lungs can contribute to persistently elevated PVR in PPHN. Previous studies demonstrated that chronic intra‐uterine pulmonary hypertension impairs lung angiogenesis and causes lung hypoplasia (Grover et al. 2005). VEGF is a critical downstream target of HIF‐1*α* (Semenza 2005). Adult onset pulmonary hypertension is characterized by plexiform lesions secondary to dysregulated proliferation of pulmonary arterial endothelial cells, resulting in vascular occlusion (Tuder et al. 2001; Fijalkowska et al. 2010). Immuno‐histochemical analysis of these plexiform lesions shows increased levels of HIF‐1*α* and increased VEGF mRNA (Tuder et al. 2001). This suggests that the lesions arise from a disordered angiogenesis resulting from pathological activation of HIF‐1*α* – VEGF pathway (Tuder et al. 2001). In our study, we observed a decrease in VEGF levels in PPHN‐PAEC along with decreased cell migration and in vitro tube formation. When HIF‐1*α* was knocked down in PPHN‐PAEC there was a significant increase in VEGF levels, resulting in improved tube formation and migration in PPHN‐PAEC, which further supports the changes in VEGF. Though contrary to previous studies in hypoxia induced angiogenesis, our findings suggest that excess levels of HIF‐1 *α* may exert a negative regulatory effect on angiogenesis in normoxia, in contrast to effects observed during hypoxia. We hypothesize that the increase in ROS secondary to HIF‐1*α* activation may in part mediate the impairment of angiogenesis in PPHN‐PAEC (Wedgwood et al. 2005, 2015; Teng et al. 2009). Another possible reason is the difference in developmental stage of the affected lung between PPHN and adult PAH as suggested by our previous reports that PAEC in PPHN are apoptosis‐prone as opposed to apoptosis‐resistant and proliferative PAEC observed in adult PAH (Tuder et al. 2001; Teng et al. 2011). When fetal cells are under low oxygen tension, HIF‐1*α* mediated VEGF release facilitates angiogenesis in the lung. In our studies, when PPHN cells were cultured under normoxic conditions, excess levels of HIF‐1*α* no longer increased the VEGF levels. We hypothesize that the presence of hypoxia normally contributes to HIF‐1a mediated activation of VEGF transcription, which did not occur in the normoxic PPHN cells. The possible additive effects of hypoxia and HIF‐1*α* on VEGF transcription require further studies.

In adult pulmonary arterial hypertension, an increase in mitochondrial hydrogen peroxide levels were shown to induce HIF‐1*α*, which then leads to a metabolic switch to glycolysis by activating glycolytic genes while simultaneously suppressing the electron transport chain (ETC) activity (Liu et al. 2004). Hek‐II is an isoform of hexokinase that phosphorylates hexoses (e.g., glucose to glucose‐6‐phosphate) aerobically to produce ATP. We previously reported that there is a decrease in ATP generation in PPHN‐PAEC, compared to control PAEC (Afolayan et al. 2016). We observed a HIF‐1*α*–dependent increase in the levels of Hek‐II in PPHN‐PAEC in this study.

The in vivo significance of increased HIF‐1*α* signaling to pulmonary vascular remodeling remains unclear. Pharmacologic inhibition of HIF‐1*α* using digoxin or inhibition of glycolysis with agents such as dichloro‐acetate may offer insights into the role of these metabolic alterations in the impaired postnatal adaptation in PPHN (Koulmann et al. 2006; Zhang et al. 2008; Yoshida et al. 2010). These studies may offer future therapeutic potential for improving postnatal adaptation in PPHN.

## Conflict of Interest

Authors do not have any financial ties to products in the study or potential/perceived conflicts of interest.

## References

[phy213986-bib-0001] Afolayan, A. J. , A. Eis , R.‐J. Teng , I. Bakhutashvili , S. Kaul , J. M. Davis , et al. 2012 Decreases in manganese superoxide dismutase expression and activity contribute to oxidative stress in persistent pulmonary hypertension of the newborn. Am. J. Physiol. Lung Cell. Mol. Physiol. 303:L870–L879. 22962015 10.1152/ajplung.00098.2012 PMC3517675

[phy213986-bib-0002] Afolayan, A. J. , A. Eis , M. Alexander , T. Michalkiewicz , R. J. Teng , S. Lakshminrusimha , et al. 2016 Decreased endothelial nitric oxide synthase expression and function contribute to impaired mitochondrial biogenesis and oxidative stress in fetal lambs with persistent pulmonary hypertension. Am. J. Physiol. Lung Cell. Mol. Physiol. 310:L40–L49. 26519208 10.1152/ajplung.00392.2014 PMC4698434

[phy213986-bib-0003] Bishop, T. , and P. J. Ratcliffe . 2015 HIF hydroxylase pathways in cardiovascular physiology and medicine. Circ. Res. 117:65–79. 26089364 10.1161/CIRCRESAHA.117.305109 PMC4501273

[phy213986-bib-0004] Bonnet, S. , E. D. Michelakis , C. J. Porter , M. A. Andrade‐Navarro , B. Thébaud , S. Bonnet , et al. 2006 An abnormal mitochondrial‐hypoxia inducible factor‐1alpha‐Kv channel pathway disrupts oxygen sensing and triggers pulmonary arterial hypertension in fawn hooded rats: similarities to human pulmonary arterial hypertension. Circulation 113:2630–2641. 16735674 10.1161/CIRCULATIONAHA.105.609008

[phy213986-bib-0005] Brennan, L. A. , R. H. Steinhorn , S. Wedgwood , E. Mata‐Greenwood , E. A. Roark , J. A. Russell , et al. 2003 Increased superoxide generation is associated with pulmonary hypertension in fetal lambs: a role for NADPH oxidase. Circ. Res. 92:683–691. 12609968 10.1161/01.RES.0000063424.28903.BB

[phy213986-bib-0006] Fijalkowska, I. , W. Xu , S. A. A. Comhair , A. J. Janocha , L. A. Mavrakis , B. Krishnamachary , et al. 2010 Hypoxia inducible‐factor1alpha regulates the metabolic shift of pulmonary hypertensive endothelial cells. Am. J. Pathol. 176:1130–1138. 20110409 10.2353/ajpath.2010.090832 PMC2832136

[phy213986-bib-0007] Fox, W. W. , M. H. Gewitz , R. Dinwiddie , W. H. Drummond , and G. J. Peckham . 1977 Pulmonary hypertension in the perinatal aspiration syndromes. Pediatrics 59:205–211. 556842

[phy213986-bib-0008] Grover, T. R. , T. A. Parker , V. Balasubramaniam , N. E. Markham , and S. H. Abman . 2005 Pulmonary hypertension impairs alveolarization and reduces lung growth in the ovine fetus. Am. J. Physiol. Lung Cell. Mol. Physiol. 288:L648–L654. 15579625 10.1152/ajplung.00288.2004

[phy213986-bib-0009] Hu, C.‐J. , L.‐Y. Wang , L. A. Chodosh , B. Keith , and M. C. Simon . 2003 Differential roles of hypoxia‐inducible factor 1 (HIF‐1) and HIF‐2 in hypoxic gene regulation. Mol. Cell. Biol. 23:9361–9374. 14645546 10.1128/MCB.23.24.9361-9374.2003 PMC309606

[phy213986-bib-0010] Konduri, G. G. , J. Ou , Y. Shi , and K. A. Jr Pritchard . 2003 Decreased association of HSP90 impairs endothelial nitric oxide synthase in fetal lambs with persistent pulmonary hypertension. Am. J. Physiol. Heart Circ. Physiol. 285:H204–H211. 12663260 10.1152/ajpheart.00837.2002

[phy213986-bib-0011] Konduri, G. G. , A. Solimano , G. M. Sokol , J. Singer , R. A. Ehrenkranz , N. Singhal , et al. 2004 A randomized trial of early versus standard inhaled nitric oxide therapy in term and near‐term newborn infants with hypoxic respiratory failure. Pediatrics 113:559–564. 14993550 10.1542/peds.113.3.559

[phy213986-bib-0012] Konduri, G. G. , I. Bakhutashvili , A. Eis , and K. Jr Pritchard . 2007a Oxidant stress from uncoupled nitric oxide synthase impairs vasodilation in fetal lambs with persistent pulmonary hypertension. Am. J. Physiol. Heart Circ. Physiol. 292:H1812–H1820. 17142346 10.1152/ajpheart.00425.2006

[phy213986-bib-0013] Konduri, G. G. , B. Vohr , C. Robertson , G. M. Sokol , A. Solimano , J. Singer , et al. 2007b Early inhaled nitric oxide therapy for term and near‐term newborn infants with hypoxic respiratory failure: neurodevelopmental follow‐up. J. Pediatr. 150:240.e1. 10.1016/j.jpeds.2006.11.06 PMC1906720 17307536

[phy213986-bib-0014] Koulmann, N. , V. Novel‐Chaté , A. Peinnequin , R. Chapot , B. Serrurier , N. Simler , et al. 2006 Cyclosporin a inhibits hypoxia‐induced pulmonary hypertension and right ventricle hypertrophy. Am. J. Respir. Crit. Care Med. 174:699–705. 16799071 10.1164/rccm.200512-1976OC

[phy213986-bib-0015] Liu, L. , L. Liping , and M. C. Simon . 2004 Regulation of transcription and translation by hypoxia. Cancer Biol. Ther. 3:492–497. 15254394 10.4161/cbt.3.6.1010

[phy213986-bib-0016] Marín‐Hernández, A. , J. C. Gallardo‐Perez , S. J. Ralph , S. Rodriguez‐Enriquez , R. Moreno‐Sanchez . 2009 HIF‐1alpha modulates energy metabolism in cancer cells by inducing over‐expression of specific glycolytic isoforms. Mini Rev. Med. Chem. 9:1084–1101. 19689405 10.2174/138955709788922610

[phy213986-bib-0017] Park, A. M. , T. A. Sanders , and E. Maltepe . 2010 Hypoxia‐inducible factor (HIF) and HIF‐stabilizing agents in neonatal care. Semin. Fetal Neonatal. Med. 15:196–202. 20599462 10.1016/j.siny.2010.05.006 PMC2924157

[phy213986-bib-0019] Rehman, J. , and S. L. Archer . 2010 A proposed mitochondrial‐metabolic mechanism for initiation and maintenance of pulmonary arterial hypertension in fawn‐hooded rats: the warburg model of pulmonary arterial hypertension. Adv. Exp. Med. Biol. 661:171–185. 20204730 10.1007/978-1-60761-500-2_11

[phy213986-bib-0020] Resnik, E. R. , J. M. Herron , S.‐C. Lyu , and D. N. Cornfield . 2007 Developmental regulation of hypoxia‐inducible factor 1 and prolyl‐hydroxylases in pulmonary vascular smooth muscle cells. Proc. Natl. Acad. Sci. USA 104:18789–18794. 18000055 10.1073/pnas.0706019104 PMC2141855

[phy213986-bib-0021] Rey, S. , and G. L. Semenza . 2010 Hypoxia‐inducible factor‐1‐dependent mechanisms of vascularization and vascular remodelling. Cardiovasc. Res. 86:236–242. 20164116 10.1093/cvr/cvq045 PMC2856192

[phy213986-bib-0022] Scortegagna, M. , K. Ding , Y. Oktay , et al. 2003 Multiple organ pathology, metabolic abnormalities and impaired homeostasis of reactive oxygen species in Epas1‐/‐ mice. Nat. Genet. 35:331–340. 14608355 10.1038/ng1266

[phy213986-bib-0023] Semenza, G. L. 2003 Targeting HIF‐1 for cancer therapy. Nat. Rev. Cancer 3:721–732. 13130303 10.1038/nrc1187

[phy213986-bib-0024] Semenza, G. L. 2005 Involvement of hypoxia‐inducible factor 1 in pulmonary pathophysiology. Chest 128:592S–594S. 10.1378/chest.128.6_suppl.592S 16373853

[phy213986-bib-0025] Teng, R.‐J. , A. Eis , I. Bakhutashvili , N. Arul , and G. G. Konduri . 2009 Increased superoxide production contributes to the impaired angiogenesis of fetal pulmonary arteries with in utero pulmonary hypertension. Am. J. Physiol. Lung Cell. Mol. Physiol. 297:L184–L195. 19429773 10.1152/ajplung.90455.2008 PMC2711810

[phy213986-bib-0026] Teng, R.‐J. , T.‐J. Wu , C. G. Bisig , A. Eis , K. A. Pritchard , and G. G. Konduri . 2011 Nitrotyrosine impairs angiogenesis and uncouples eNOS activity of pulmonary artery endothelial cells isolated from developing sheep lungs. Pediatr. Res. 69:112–117. 21057377 10.1203/PDR.0b013e318204dcb8 PMC3086583

[phy213986-bib-0027] Teng, R.‐J. , J. Du , A. J. Afolayan , A. Eis , Y. Shi , and G. G. Konduri . 2013 AMP kinase activation improves angiogenesis in pulmonary artery endothelial cells with in utero pulmonary hypertension. Am. J. Physiol. Lung Cell. Mol. Physiol. 304:L29–L42. 23103561 10.1152/ajplung.00200.2012 PMC3543642

[phy213986-bib-0028] Tuder, R. M. , M. Chacon , L. Alger , J. Wang , L. Taraseviciene‐Stewart , Y. Kasahara , et al. 2001 Expression of angiogenesis‐related molecules in plexiform lesions in severe pulmonary hypertension: evidence for a process of disordered angiogenesis. J. Pathol. 195:367–374. 11673836 10.1002/path.953

[phy213986-bib-0029] Walsh‐Sukys, M. C. , J. E. Tyson , L. L. Wright , C. R. Bauer , S. B. Korones , D. K. Stevenson , et al. 2000 Persistent pulmonary hypertension of the newborn in the era before nitric oxide: practice variation and outcomes. Pediatrics 105:14–20. 10617698 10.1542/peds.105.1.14

[phy213986-bib-0031] Wedgwood, S. , R. H. Steinhorn , M. Bunderson , J. Wilham , S. Lakshminrusimha , L. A. Brennan , et al. 2005 Increased hydrogen peroxide downregulates soluble guanylate cyclase in the lungs of lambs with persistent pulmonary hypertension of the newborn. Am. J. Physiol. Lung Cell. Mol. Physiol. 289:L660–L666. 15937064 10.1152/ajplung.00369.2004 PMC2733241

[phy213986-bib-0032] Wedgwood, S. , S. Lakshminrusimha , P. T. Schumacker , and R. H. Steinhorn . 2015 Hypoxia inducible factor signaling and experimental persistent pulmonary hypertension of the newborn. Front. Pharmacol. 6:47. 25814954 10.3389/fphar.2015.00047 PMC4356070

[phy213986-bib-0033] Yoshida, T. , H. Zhang , T. Iwase , J. Shen , G. L. Semenza , and P. A. Campochiaro . 2010 Digoxin inhibits retinal ischemia‐induced HIF‐1alpha expression and ocular neovascularization. FASEB J. 24:1759–1767. 20065104 10.1096/fj.09-145664 PMC2874483

[phy213986-bib-0034] Zhang, H. , D. Z. Qian , Y. S. Tan , K. Lee , P. Gao , Y. R. Ren , et al. 2008 Digoxin and other cardiac glycosides inhibit HIF‐1alpha synthesis and block tumor growth. Proc. Natl. Acad. Sci. USA 105:19579–19586. 19020076 10.1073/pnas.0809763105 PMC2604945

